# Effect of Different Cooling Strategies on Surface Quality and Power Consumption in Finishing End Milling of Stainless Steel 316

**DOI:** 10.3390/ma14040903

**Published:** 2021-02-14

**Authors:** Adel T. Abbas, Saqib Anwar, Elshaimaa Abdelnasser, Monis Luqman, Jaber E. Abu Qudeiri, Ahmed Elkaseer

**Affiliations:** 1 Mechanical Engineering Department, College of Engineering, King Saud University, P.O. Box 800, Riyadh 11421, Saudi Arabia; monislugman9@gmail.com; 2 Industrial Engineering Department, College of Engineering, King Saud University, P.O. Box 800, Riyadh 11421, Saudi Arabia; sanwar@ksu.edu.sa; 3 Department of Production Engineering and Mechanical Design, Faculty of Engineering, Port Said University, Port Fuad 42526, Egypt; Alshymaa.gamal@eng.psu.edu.eg (E.A.); ahmed.elkaseer@kit.edu (A.E.); 4 Mechanical Engineering Department, College of Engineering, United Arab Emirates University, Al Ain 15551, United Arab Emirates; jqudeiri@uaeu.ac.ae; 5 Institute for Automation and Applied Informatics, Karlsruhe Institute of Technology, 76344 Karlsruhe, Germany

**Keywords:** stainless steel 316, finishing end milling operation, cooling strategies, dry condition, MQL, nanoparticles based cutting fluids, surface roughness, power consumption

## Abstract

In this paper, an experimental investigation into the machinability of AISI 316 alloy during finishing end milling operation under different cooling conditions and with varying process parameters is presented. Three environmental-friendly cooling strategies were utilized, namely, dry, minimal quantity lubrication (MQL) and MQL with nanoparticles (Al_2_O_3_), and the variable process parameters were cutting speed and feed rate. Power consumption and surface quality were utilized as the machining responses to characterize the process performance. Surface quality was examined by evaluating the final surface roughness and surface integrity of the machined surface. The results revealed a reduction in power consumption when MQL and MQL + Al_2_O_3_ strategies were applied compared to the dry case by averages of 4.7% and 8.6%, respectively. Besides, a considerable reduction in the surface roughness was noticed with average values of 40% and 44% for MQL and MQL + Al_2_O_3_ strategies, respectively, when compared to the dry condition. At the same time, the reduction in generated surface roughness obtained by using MQL + Al_2_O_3_ condition was marginal (5.9%) compared with using MQL condition. Moreover, the results showed that the improvement obtained in the surface quality when using MQL and MQL + Al_2_O_3_ coolants increased at higher cutting speed and feed rate, and thus, higher productivity can be achieved without deteriorating final surface quality, compared to dry conditions. From scanning electron microscope (SEM) analysis, debris, furrows, plastic deformation irregular friction marks, and bores were found in the surface texture when machining under dry conditions. A slight smoother surface with a nano-polishing effect was found in the case of MQL + Al_2_O_3_ compared to the MQL and dry cooling strategies. This proves the effectiveness of lubricant with nanoparticles in reducing the friction and thermal damages on the machined surface as the friction marks were still observed when machining with MQL comparable with the case of MQL + Al_2_O_3_.

## 1. Introduction

Molybdenum-bearing austenitic stainless steels are types of stainless steel alloys that are more resistant to general corrosion and pitting/crevice corrosion than the conventional chromium-nickel austenitic stainless steels [1]. In addition to excellent corrosion resistance and strength properties, these alloys also offer higher creep, stress-to-rupture and tensile strength at elevated temperatures [2]. Stainless steel 316 material, as part of the molybdenum-bearing austenitic stainless steels group, is ubiquitously used in the chemical and petrochemical industry, in food processing, medical devices, pharmaceutical equipment, in potable water, wastewater treatment, in marine applications, and architectural applications near the seashore or in urban areas [3].

However, the machining of these materials is quite problematic. It is because of high strength, low thermal conductivity, and the tendency of work hardening [2,4]. These properties make stainless steel 316 material tend to machine with higher cutting force, higher generated cutting temperature, and built-up edge formation, which negatively affected the machined surface quality and tool life. Moreover, the high toughness of Stainless steel 316 material results in unpropitious chip breakage which causes burr formation and accelerates tool wear. It is quite worthy of mentioning that optimum cutting speed and feed rate are also prominent parameters for tool life extension [5].

Numerous researches have been conducted to overcome these problems. Verma [6] investigated the effect of process parameters on surface quality when machining stainless steel 316 by measured surface roughness and found that higher feed rate and depth of cut resulted in a rougher surface while increasing cutting speed led to a smoother surface. El-Hossein et al. [7] experimented on the effect of multi-layered carbide inserts in milling of stainless steel and reported that the tool wears dramatically improved with an increase in cutting speed and by reducing the feed rate. Liew and Ding in their research compared the wear resistance of TiAlN Physical Vapor Deposition (PVD) coated and uncoated carbide inserts during the milling of AISI 420 stainless steel material; they observed an enhanced abrasive wear resistance and prevention of chipping with the use of coated carbide end mills. Moreover, the research also depicted that the use of cutting fluid with reinforced metallic nanoparticles would reduce cutting tool failure problems [8].

Cutting fluids are enormously being utilized in metal removal techniques to improve surface finish, enhance tool life, productivity, and integrity [9,10,11,12]. In general, heat is generated during machining operation due to plastic deformation in the shear zones and friction. Consequently, high-temperature gradients are developed, resulting in tool wear [13], which, in turn, causes shattering tool failure. In order to overcome such catastrophic failures, numerous cooling techniques like flood coolant [14] and high-pressure coolant systems [15] are used during the milling process [16]. However, due to an increase in the production cost for these techniques, an alternative Minimum Quantity Lubrication (MQL) technique, especially for high-speed milling, has been developed [17]. In the MQL technique, a small amount of cooling fluid, less than those used in conventional cooling strategy is mixed with compressed air to form a spray of micro drops crushed in the cutting region [18,19].

In order to add extend its functionality, nanofluids are produced by incorporating metallic nanoparticles such as Aluminum Oxide (Al_2_O_3_), Carbon nanotubes, Graphene, Diamond, Titanium Dioxide (TiO_2_), and Molybdenum Disulphide (MoS_2_) to the cooling fluids. The addition of these metallic nanoparticles improves the lubrication effect and thermal conductivity of the cutting fluid, which consequently enriches the performance of the MQL technique. Shen et al. [20], in their research, utilized the MQL technique by adding Al_2_O_3_ and diamond nanoparticles in water and found enhanced surface roughness, force reduction, and decreased workpiece burning. Additionally, they also depicted that the MQL technique shows a dramatic reduction in friction and force due to the addition of MoS_2_ metallic nanoparticles to the cutting fluids [21]. Rahmati et al. [22] applied the MQL technique with MoS_2_ as a reinforced metallic nanoparticle and investigated the effect of MoS_2_ nanoparticles on the morphology and surface quality of the machining sample; it was found that the presence of MoS_2_ enhanced the machined surface quality. Another research found that the addition of nano-diamond in the MQL technique would suggestively increase the tool life, decrease thrust force and torque due to enhanced lubrication and cooling [23]. Setti et al. [24] utilized Al_2_O_3_ nanoparticles in the MQL technique for machining Ti6Al4V; it was depicted that the addition of Al_2_O_3_ enhanced surface integrity and reduced coefficient of friction due to the prevention of tribofilm on the machined surface. Furthermore, Alberts et al. [25] incorporated graphite nano-platelet into the cutting fluid and found lower cutting force and improved surface finish. Li et al. [26] indicated that graphene nanoparticle (GPNP) incorporation strengthens the cutting fluid performance, which dramatically enhanced the cooling and lubricating performance during MQL grinding operation.

Although the application of nanofluids has shown potential in improving the machining responses of different materials, some drawbacks have been also highlighted such as the negative effect of added nanoparticles on the machined surfaces due to the uncontrolled scratching effect [27]. In this context, the aim of this research is to comparatively assess the performance of different cooling strategies under a range of cutting parameters. In particular, an experimental investigation was carried out by a number of finishing end milling trials to examine the machinability of stainless steel 316 alloy under different cooling conditions and process parameters. The trend these days is to conduct machining processes in dry conditions to reduce the overall machining costs and to take advantage of it being an environmentally friendly condition. The three types of coolant used in the presented work are dry, minimum quantity lubrication (MQL), and MQL with nanoparticles (MQL + Al_2_O_3_). The effect of process parameters, namely, feed rate and cutting speed, and the aforementioned three different cooling strategies on surface quality and power consumption are carried out. Besides, surface integrity was examined using a scanning electron microscope for machined specimens.

## 2. Materials and Methods

### 2.1. Material Specifications

As formerly stated, due to their distinct mechanical properties, stainless steel 316 has many uses in different industries such as the aircraft industry, chemical industry, pump shafts, medical instruments, and food preparation equipment. The material used in this research study is “stainless steel 316”. The chemical composition of this material is shown in Table 1, while Table 2 shows the mechanical properties of this alloy.

### 2.2. Machining of Test Specimens

**Figure 1:**
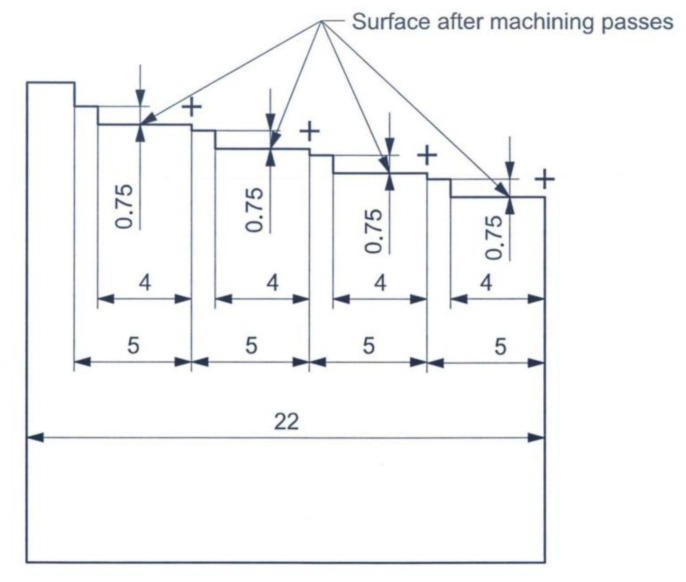
Drawing for workpiece illustrating machining passes, (all dimensions are in mm).

**Figure 2:**
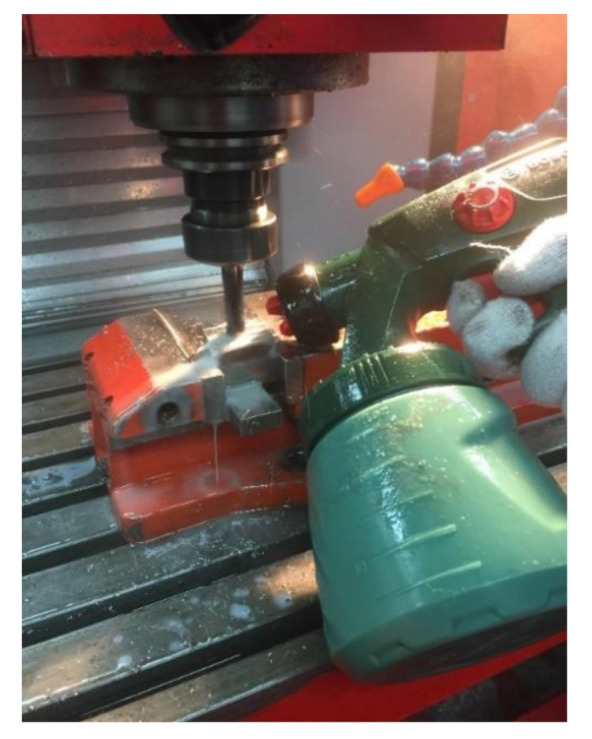
Test rig for machining the workpieces.

Conventional vertical milling machining “Emco Mill C40” (Emco, Salzburg, Austria) was used for machining the workpieces with the following dimensions: length = 35 mm, width = 22 mm and height = 35 mm. The spindle has a power of 13 KW and steeples revolution of 10–5000 rpm. The table has steeples feed rates of 10–5000 mm/min. The end-mill is manufactured by Sandvik (Stockholm, Sweden) with the ordering code: 1P240-1200-XA 1630. End-mill is made of solid tungsten carbide with 12 mm diameter, four flutes and a cutting length of 24 mm. This tool was designed for providing high-quality surface finishes with efficient material removal rates. It is commonly used for all types of materials from stainless steel to titanium alloys. The drawing of the workpiece showing machining passes in the test runs is shown in Figure 1, while the test ring for conducting the machining tests is shown in Figure 2.

### 2.3. Measuring Systems

**Figure 3:**
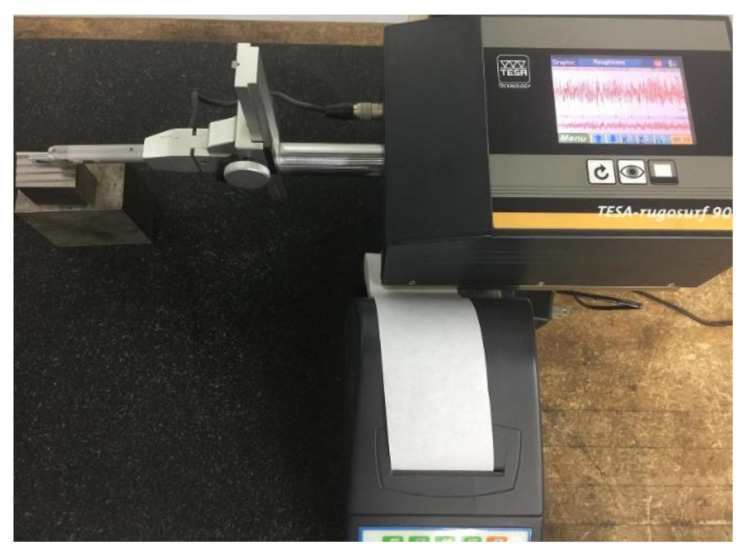
Test rig for measuring surface roughness.

**Figure 4:**
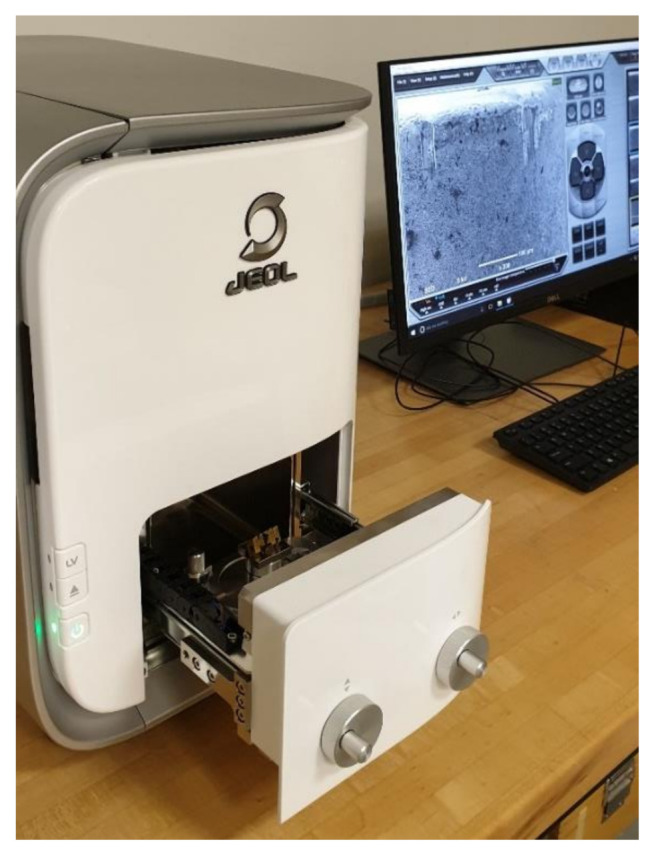
Scanning electron microscope setup for observing surface morphology.

A TESA (TESA, Bugnon, Switzerland) surface roughness tester, type” Rugosurf 90-G”, is used for the evaluation of the machined surface roughness. The test rig for measuring the surface roughness is shown in Figure 3. A tabletop Scanning Electron Microscope (SEM) type: JCM 6000 Plus from JEOL, Tokyo, Japan, as shown in Figure 4, was used to characterize the surface morphology of the milled samples. All surface roughness measurements were taken in a longitudinal direction parallel to the feed rate direction with a cut-off of 0.8 mm, and 5 measurements of 15 mm evaluation length were recorded for each sample.

The power supply of the machine was connected to two measuring power meters Type: Tactix 403057 (Tactix, Beijing, China); the first one was used to measure the voltage and the second one for measuring the current. According to the datasheet of the machine, it is a balanced three-phase load. The power is measured as follows: One ammeter is connected to measure one-line current (I) and the line to line voltage is measured by a voltmeter (V). The load power factor is taken from the datasheet. The accuracy of the equipment is 1%. The reading was taken three times in each milling test and the power consumption was calculated according to Equation (1).

*Power* = *Voltage* × *Current* × √3 cos*ø* = *Watt* (1)

### 2.4. Test Procedures

The experimental work in this paper included (24) milling runs. Constant cutting conditions are listed in Table 3. The test runs were divided into three groups, each group was subjected to a different type of cooling strategy. The test runs are presented in Table 4 in which one-factor at a time method was used, and the main variable parameters are namely speed (V) in [mm/ min] and feed rate (f) in [mm/min]. Due to its limited effect, the axial depth of cut is fixed at 0.75 mm, and the radial depth of cut is fixed at 4 mm. Whereas the speed is varied at different levels of 30, 50, 60, 90, and 120 m/min, and the feed rate at the values of 25, 50, 100, and 125 mm/min were applied for a constant cutting length of 35 mm in each trial. This group of variables is applied three times with different types of coolants, namely, dry cutting, an MQL of Sunflower oil, and MQL of Sunflower oil mixed with Aluminum Oxide (Al_2_O_3_) nanoparticles (MQLNF). It is worth mentioning that previous studies [28,29] have shown that Al_2_O_3_ nanoparticles are non-toxic in nature.

### 2.5. Cooling Conditions

The first eight tests were done without using any type of cutting fluid, i.e., under dry conditions.

The second group of eight tests was carried out using an MQL of Sunflower oil, possessing physicochemical properties of vegetable-based oils such as a Kinematic viscosity: 40 1C (cSt): 40.05, viscosity index: 206, Flashpoint (0 °C): 252 and Pour point (0 °C): −12.00.

**Figure 5:**
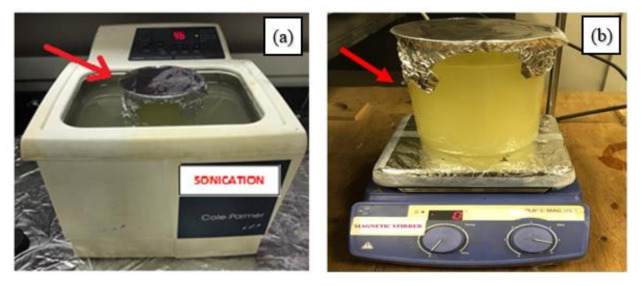
The preparation of the Minimum Quantity Lubrication (MQL)with nanoparticles (**a**) sonication and (**b**) magnetic stirring process of the nanofluid.

The third group of eight tests was carried out using MQL of Sunflower oil mixed with nano aluminum oxide Al_2_O_3_. The concentrations of nanoparticles in vegetable base oils were 0.2 wt.% (as recommend in the open literature, and to avoid any clogging while preparing the nanofluid). The mixture was then ultra-sonicated in high frequency 40 kHz sonicator (Cole-Parmer 8893), supplied by Cole-Parmer, Chicago, IL, USA, for about 60 min, and then, the fluid was stirred for 30 min using a magnetic stirrer as seen in Figure 5. The aforementioned preparation steps were repeated as a cyclic process till the Nanoparticles were uniformly dispersed in the vegetable oil. Moreover, it is quite worthy to mention that the prepared Nano-fluid was stable without any settlement of particles during the machining process. The MQL and MQL with Al_2_O_3_ nanoparticles were operated using a Bosch spray pump (PFS1000, Bosch, Berlin, Germany) having a power input of 410 W and adjustable flow rate 0–100 mL/min. A flow rate of 2 mL/min was adopted for all the trials.

The details of the test runs and measurements are recorded in Table 4.

## 3. Results and Discussion

### 3.1. Effect of Cooling Strategies on Power Consumption

**Figure 6:**
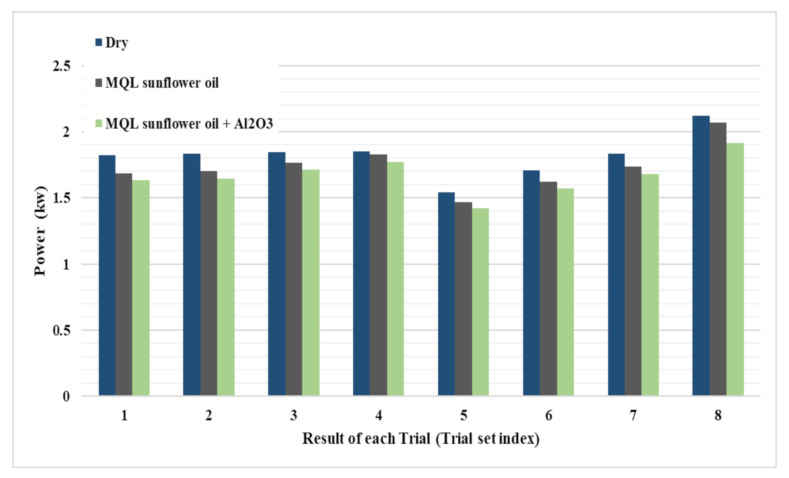
Comparison between the three cooling types (Dry, MQL and MQL + Al_2_O_3_) on power consumption during finishing end milling of stainless steel 316.

Figure 6 presents a comparison between the three types of coolant in measured power consumption for each trail at the same cutting parameters. It was found that machining under dry condition consumed a higher power than the other two cooling conditions while cutting under MQL with nanoparticles gave a lowest power consumption. The decrease in power consumption in MQL and MQL + Al_2_O_3_ compared to dry case was 4.7% and 8.6%, respectively. This is due to the effectiveness of lubrication by MQL in reducing the friction of chip-tool interface which results in reduction on cutting force [30], thus less power was consumed compared with the case of dry machining. Besides, using MQL in cutting prevents built-up edge formation and rapid tool wear which lead to a reduction in cutting force and power consumption [31]. Moreover, some studies observed the benefits of MQL in improving chip thickness ratio as the chip thickness decrease when using MQL compared to the dry condition [32]. This means that resistance to cut decreases by using MQL, thus less power consumption occurs. Cutting with MQL + Al_2_O_3_ decreased the power consumption even more than the case of using MQL; see Figure 6. Bai et al. [30] reported that the addition of nanoparticles to MQL oil increases the viscosity of the fluid which formed a thin layer of oil film on the friction surface of the workpiece. This can improve the oil adsorption effect, thus improving the lubrication performance and helping in reducing friction and wear compared to the case of using pure oil. Moreover, increasing viscosity of oil by the addition of nanoparticles prevents the flow of fluid away from cutting zone and helps in maintaining fluid for a long time in the cutting zone which leads to a reduction in friction between the workpiece and flank face of the tool and the generated chips and the rake face of the cutting tool and thus reduces tool wear. This reduction in friction of chip-tool interface and tool wear decreases cutting force and is thus associated with less power consumption.

**Figure 7:**
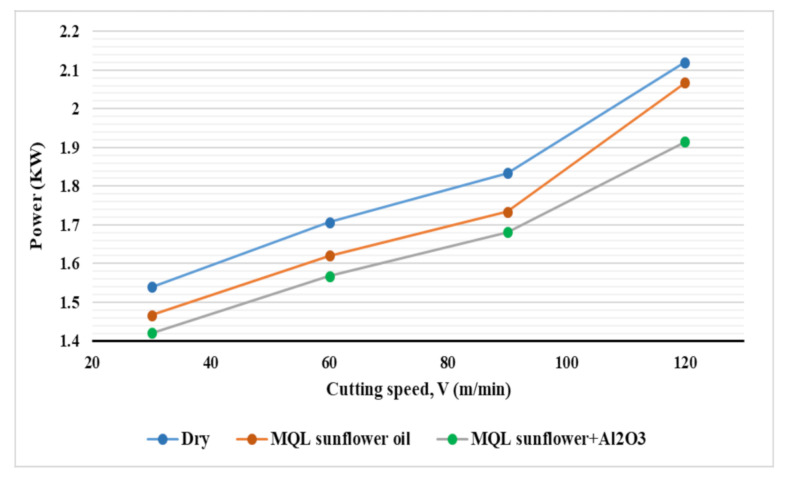
Effect of cooling strategies on power consumption with different cutting speeds.

**Figure 8:**
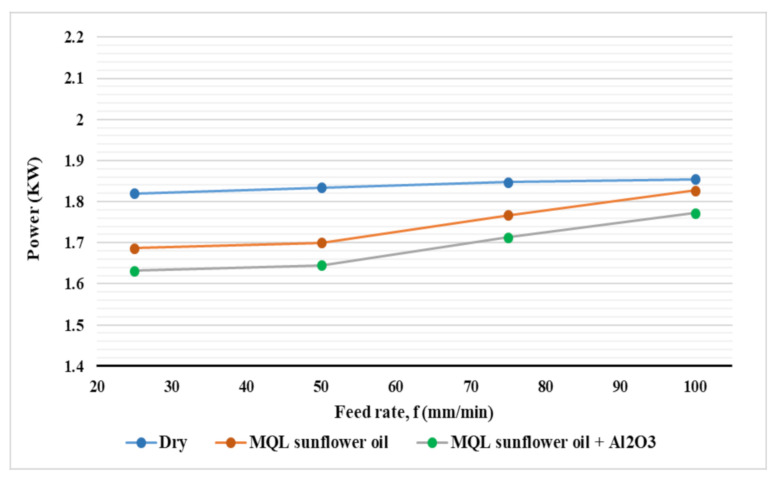
Effect of cooling strategies on power consumption with different feed rates.

Figure 7 and Figure 8 show the effect of cutting speed and feed rate, respectively, on power consumption for the three types of coolant. According to the effect of cutting speed on power consumption (see Figure 7), an obvious increase in power consumption with increasing cutting speed for all coolant types was found. When a higher cutting speed is applied, a larger rotational movement of the spindle is applied, and hence, more power is consumed [31]. With regards to the effect of feed rate on power consumption (Figure 8), a lower influence of feed rate on power consumption compared to the influence of cutting speed was observed. An increase in power consumption with increasing feed rate for the three types of coolant was found. This is due to the higher velocity of axes motor movement with a higher feed rate which leads to an increase in power consumption [31]. Moreover, the power consumed due to cutting is increased at a higher feed rate due to larger chip thickness (chip load) which increases the resistance to cutting and increases power consumption. In addition, it was noticed that at a lower feed rate (25 mm/min), the difference between the effectiveness of the three cooling systems was higher than that obtained at a higher feed rate (100 mm/min). At dry machining, the neglectable influence of the feed rate was found on power consumption as compared with the other two cases.

### 3.2. Effect of Cooling Strategies on Surface Roughness

**Figure 9:**
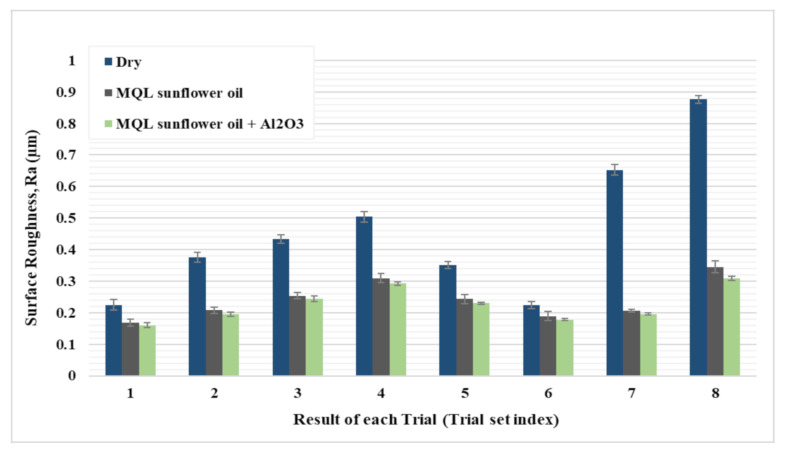
Comparison between the three coolant types (Dry, MQL, and MQL+ Al_2_O_3_) on measured surface roughness.

Figure 9 shows a comparison between the three types of coolant in measured surface roughness under the same cutting conditions. A substantial improvement of surface quality when using lubricant (MQL and MQL with nanoparticles) was observed compared to dry cutting for all trials while a further slight decrease in surface roughness was found when using MQL with nanoparticles compared with using MQL with pure oil. In particular, the improvements in surface roughness in the cases of MQL and MQL + Al_2_O_3_ were found to be 40% and 44%, respectively, compared to the dry case. This refers to the benefits of mist oil in lubricating the area of the cutting zone and reduce the friction between the tool and workpiece [31]. Consequently, less adhesion effect, minimum built-up edge formation, and less wear result in the improvement of surface quality. Moreover, the decrease in cutting force due to friction reduction decreases the fluctuation of forces and leads to a smoother surface [30]. This is besides the effectiveness of lubricant in cooling which helps in decreasing generated cutting temperature and proper thermal evacuation, which reduces thermal damage on the workpiece and cutting tool and results in a reduction in surface roughness [32]. Especially, Al_2_O_3_ particles have a high thermal conductivity which helps in improving heat transfer of oil and reducing the cutting temperature in the cutting zone. Therefore, less thermal damage occurs on the workpiece and cutting tool [32]. Moreover, the reduction in friction and wear effect is further accelerated by the addition of nanoparticles as a result of increasing the viscosity of the oil and forming a tribo-film oil in the cutting zone [30]. Moreover, spherical nanoparticles act as a ball bearing on the workpiece surface, which positively affects surface roughness as they help in transferring sliding friction on the machined surface to rolling friction and increasing the polishing effect [32]. All the above reasons help in improving surface roughness with the addition of nanoparticles to mist oil. Therefore, the addition of nanoparticles (Al_2_O_3_) to MQL oil showed a smoother surface more than using MQL; see Figure 9.

**Figure 10:**
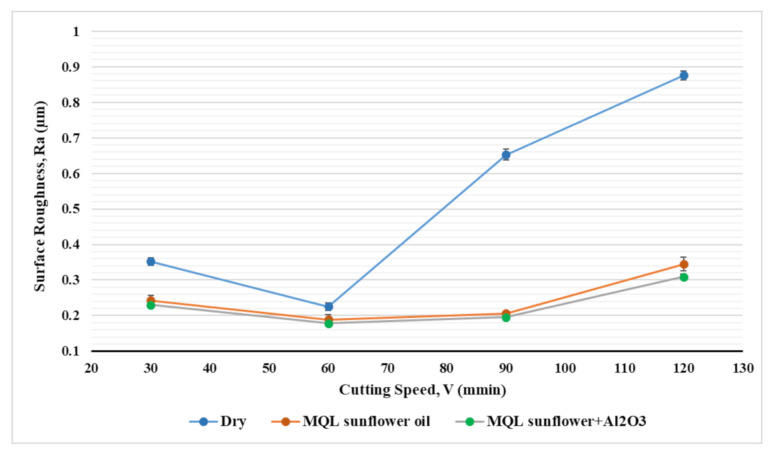
Effect of cooling strategies on surface roughness under different cutting speeds.

**Figure 11:**
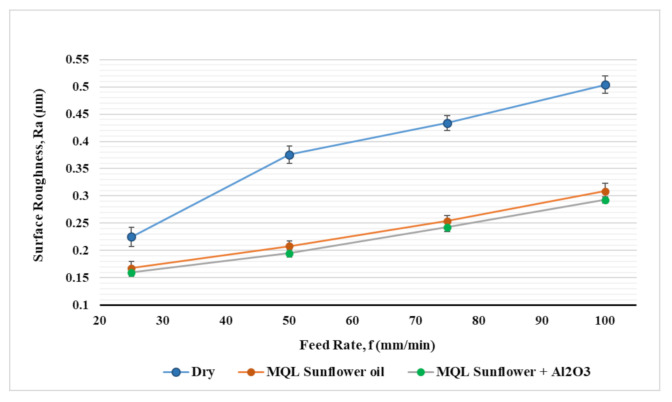
Effect of cooling strategies on surface roughness under different feed rates.

**Figure 12:**
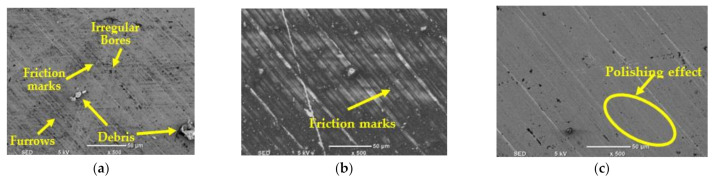
SEM of machined surface under different cooling conditions (**a**) dry, (**b**) MQL, and (**c**) MQL + Al_2_O_3_ nanoparticles at cutting speed = 50 m/min and feed rate = 100 mm/min.

Figure 10 and Figure 11 show the effect of feed rate and cutting speed on surface roughness for the three coolant types. A slight decrease in surface roughness was observed with increasing cutting speed from 30 m/min to 60 m/min followed by an increase in surface roughness when cutting speed rises from 60 m/min to 120 m/min for all coolant types; Figure 10. Increasing surface roughness with higher cutting speed is due to the increase in friction as a result of higher metal removal rate and increase the cutting temperature which led to thermal damage in the workpiece surface. This was observed obviously in the case of dry machining as higher raise was found on surface roughness by increasing cutting speed while a less influence of surface roughness was observed by increasing cutting speed in the case of using MQL and MQL + Al_2_O_3_. It was found that the reduction in surface roughness when using MQL + Al_2_O_3_ compared to dry condition was increased from 34% to 64% when cutting speed changed from 30 m/min to 120 m/min. This confirms the effectiveness of mist lubrication in the reduction in cutting temperature and friction generated by the higher cutting speed, which minimizes their negative effect on machined surface and helps to obtain a smoother surface. Concerning the effect of feed rate on surface roughness (see Figure 12), it was found that increasing the feed rate resulted in a rougher surface for all cooling conditions. Machining with the dry condition showed a higher negative influence on surface roughness by varying feed rate more than the other two cooling conditions. The improvement in surface roughness by machining with MQL + Al_2_O_3_ comparable with the dry condition was 28% when feed rate was 25 mm/min while the value increased to reach 41% improvement on surface roughness at a higher feed rate of 100 mm/min. Therefore, one can conclude that machining with MQL+ nanoparticles showed more benefits on machining with a higher feed rate; thus, it can help obtain higher productivity with a relatively smoother surface compared to dry conditions.

### 3.3. Effect of Cooling Strategies on Surface Integrity

Figure 12 presents SEM images of the machined surface under dry, MQL, and MQL + Al_2_O_3_ conditions at cutting speed of 50 m/min and 100 mm/min feed rate. Debris, furrows, plastic deformation irregular friction marks, and bores were found in the surface texture when machining under dry condition; Figure 12a. In the machined surface in the case of MQL + Al_2_O_3_, a smoother texture without plastic deformation or debris was found while friction marks were observed obviously when machining with MQL comparable with the case of MQL + Al_2_O_3_ (Figure 12b), while polishing effect was observed in the case of MQL + Al_2_O_3_ nanoparticles, Figure 12c.

**Figure 13:**
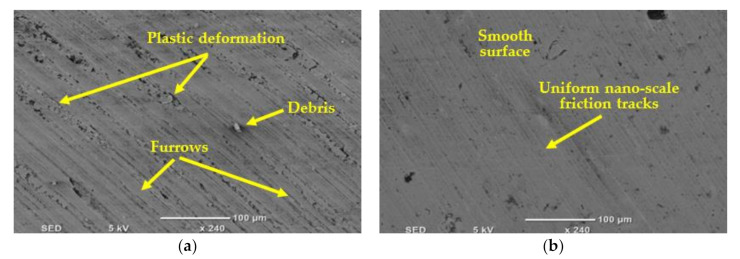
SEM of machined surface under different cooling conditions (**a**)dry and (**b**) MQL + Al_2_O_3_ nanoparticles at cutting speed = 50 m/min and feed rate = 75 mm/min.

Figure 13 shows the difference in machined surface texture between dry and MQL + Al_2_O_3_ conditions at cutting speed of 50 m/min and 75 mm/min feed rate. By looking at Figure 13a, large flaws were found in the machined surface when machining by dry condition like plastic deformation, furrows, and debris, which formed due to high friction and temperature. A smoother surface with uniform nanoscale friction tracks was found in the case of MQL + Al_2_O_3_, which reflected the polishing effect (Figure 13b) and proves the effectiveness of lubricant with nanoparticles in reducing the friction and thermal damages on the machined surface. The nano-polishing effect found in the case of MQL + Al_2_O_3_ condition is due to the bearing effect of nanoparticles on the machined surface of the workpiece [32].

**Figure 14:**
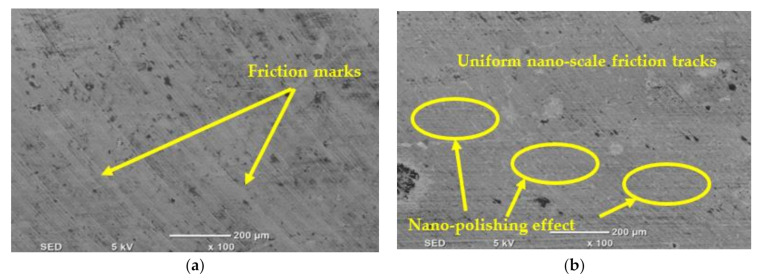
Comparison in SEM images between (**a**) MQL and (**b**) MQL + Al_2_O_3_ nanoparticles cooling conditions at cutting speed = 30 m/min and feed rate = 50 mm/min.

Figure 14 illustrates the difference in surface texture when machining by MQL and MQL + Al_2_O_3_ conditions at cutting speed = 30 m/min and feed rate = 50 mm/min. Although the difference in measured surface roughness between the two cases was very small, the SEM images presented some differences associated with friction marks, especially in the case of MQL (Figure 14a); compared with MQL + Al_2_O_3_ (Figure 14b), fewer friction marks can be detected with more uniform nano-scale friction tracks. This reflects the bearing effect of the nanoparticles when machining with MQL + Al_2_O_3_ condition compared by MQL case. This is because the nanoparticles (Al_2_O_3_) help in transferring sliding friction on the machined surface to rolling friction and increasing the polishing effect.

## 4. Conclusions

In this paper, milling tests were conducted in order to investigate the machinability of stainless steel 316 alloy under different coolant types and with varying process parameters. Dry, MQL, and MQL + Al_2_O_3_ nanofluid were the three types of coolant examined in this study, and the variables process parameters were feed rate and cutting speed. Power consumption and surface quality were examined as the quality marks in the machining trials. Machined surface quality was examined by measuring surface roughness and characterizing the machined surface texture with scanning electron microscope. The main conclusions are as follows:

- A decrease in power consumption was found by machining with MQL and MQL + Al_2_O_3_ compared to dry case by 4.7% and 8.6% in average, respectively.
- Higher improvement in surface roughness was obtained by machining with the two types of MQL lubricant conditions compared to dry condition while the difference in generated surface roughness obtained by using MQL and MQL + Al_2_O_3_ conditions was small. The improvement in surface roughness in the cases of MQL and MQL + Al_2_O_3_ found to be 40% and 44% in average, respectively, compared to dry case.
- Power consumption was found to increase with increasing cutting speed and feed rate, and the influence of the cutting speed was higher than that obtained by feed rate at all types of coolant, while at dry condition, neglectable influencing of feed rate was found on power consumption.
- It was found that improvement in surface roughness when using MQL + Al_2_O_3_ compared to dry condition was increased from 34% to 64% when cutting speed changed from 30 m/min to 120 m/min at constant value of feed rate, and this improvement was from 28% to 41% when feed rate changed from 25 mm/min to 100 mm/min at constant value of cutting speed. Therefore, the benefits of using MQL and MQL + Al_2_O_3_ coolants increased at higher cutting speed and feed rate, thus higher productivity was achieved without higher deterioration in the surface roughness compared to dry conditions.
- Adhered material, debris, furrows, plastic deformation, and bores were found in the surface texture characterized by SEM when machining with dry condition. A smoother surface with nano-polishing effect was found in the case of MQL+ Al_2_O_3_, and friction marks were observed when machining with MQL comparable with the case of MQL + Al_2_O_3_.

## Figures and Tables

**Table 1:** Chemical composition for stainless steel 316.

C.	Cr	Mn	Mo	Ni	P	S	Si	V
0.077	17.125	1.974	1.853	10.177	0.0004	0.005	0.489	0.0615

**Table 2:** Mechanical properties for stainless steel 316.

Mechanical Properties	Value
Ultimate Tensile Strength	520 N/mm^2^
0.2% Proof Strength	208 N/mm^2^
Elongation (in length 51 mm)	40%
Modulus of Elasticity	200 GPa
Modulus of Shear	82 GPa
Hardness	215 HB

**Table 3:** Constant cutting conditions applied during the whole trials.

Cutting Conditions	Value
Cutting Diameter	12 mm
Cutting Length	24 mm
Number of Flutes	4
Axial Depth	0.75 mm
Radial Depth	4 mm

**Table 4:** Test runs under different cooling conditions; depth of cut = 0.75 mm, radial depth of cut = 4 mm.

Trial #	Cutting Speed, V (m/min)	Feed Rate, f (mm/min)	Chip Load per Flute (mm/tooth)	Dry		Sunflower Oil MQL Coolant		Sunflower Oil + Nano Al_2_O_3_-Based MQL Coolant	
				Ra µm	Power KW	Ra µm	Power KW	Ra µm	Power KW
1	50	25	0.0047	0.224	1.820	0.169	1.687	0.161	1.632
2	50	50	0.0094	0.375	1.834	0.209	1.700	0.196	1.645
3	50	75	0.0141	0.436	1.847	0.257	1.767	0.243	1.713
4	50	100	0.0188	0.505	1.854	0.311	1.827	0.294	1.772
5	30	50	0.0157	0.351	1.540	0.244	1.467	0.231	1.421
6	60	50	0.0079	0.227	1.707	0.188	1.620	0.179	1.568
7	90	50	0.0052	0.657	1.834	0.207	1.734	0.197	1.681
8	120	50	0.0039	0.877	2.120	0.346	2.067	0.310	1.915

## Data Availability

The data presented in this study are available on request from the corresponding author.
